# Is human platelet lysate (hPL) the ideal candidate to substitute the foetal bovine serum for cell-therapy translational research?

**DOI:** 10.1186/s12967-021-03104-w

**Published:** 2021-10-13

**Authors:** M. Guiotto, M. O. Riehle, W. Raffoul, A. Hart, P. G. di Summa

**Affiliations:** 1 Department of Plastic, Reconstructive and Hand Surgery, Centre Hospitalier Universitaire Vaudois (CHUV), Lausanne, Switzerland; 2 Centre for the Cellular Microenvironment, University of Glasgow, Glasgow, UK; 3 Canniesburn Plastic Surgery Unit, Glasgow Royal Infirmary, Glasgow, UK

Dear Sir,

The high demand for innovative therapeutic treatments has prompted increasing efforts and resources in translational medicine in last decades. Cell therapy is an emerging strategy, which can potentially cope with challenging diseases in a variety of medical fields, including plastic surgery.

Particularly, adipose derived stem cells (ADSC) play a crucial role among other mesenchymal stem cell (MSC) candidates, considering their multi-lineage differentiation plasticity, the easiness of harvest and their abundance. ADSCs can be harvested under local anesthesia (e.g. liposuction) with minimal morbidity, which makes this cell population appealing for clinical translation [1].

To best of our knowledge, human clinical studies with ADSC in plastic surgery are limited for aesthetic purposes, particularly for volume augmentation and scar correction, while more generally MSC-therapy from different tissue sources (adipose tissue, bone marrow, skin, umbilical cord) were trialed successfully in wound healing and in severe burn patients [2, 3].

The risk posed by cell-based medicinal product depends on the cell origin, the manufacturing process (collection, selection, culture or genetic modification), the non-cellular additive components (enzymes, cytokines, medium supplements and antibiotics) and on the specific therapeutic use. This exposure can impact on the quality, safety and efficacy of the final product with a wide range of patient and healthcare personnel risks which necessarily must be assessed with a multisystemic approach.

Despite pre-clinical promising achievements, clinical translation of stem cell therapy is still limited by biosafety and ethical issues. Regarding the safety concerns, foetal bovine serum (FBS), is still the most common supplement medium for cell culture. FBS, derived from foetal bovine blood, is rich in growth factors, nutrients and hormones, promotes proliferation and maintenance of cells with limited costs. However, it increases the risk of immune reactions and exposes patients to possible viral, bacterial or prion infection. Considering a large-scale production, the manipulation with xenogeneic animal-derived components, such as FBS, can contrast with ethical/religious constrains (inhumane methods of fetal bovine blood extraction, 3R principles, religious dogmas etc.). Moreover, from a scientific point of view, the heterogeneity between the FBS batches can impact on outcomes reliability and reproducibility [4].

To limit or avoid the animal contaminants, some authors apply washing steps at the end of the FBS-cell culture, alternatively, others keep cells in autologous plasma for the 48 h before the patient administration or substitute entirely the animal sera with human serum or human platelet lysate (hPL) [2].

HPL, obtained as pooled blood from buffy coats (mainly Europe) or platelet-rich-plasma (PRP) methods (USA and Asia) (Fig. 1A, B), has been proposed as an alternative to FBS: hPL is a cell-free, protein and growth factor-enriched concentrate used as additive of growth medium for in vitro cell culture and expansion protocols in clinical-grade cell-based products [4].

**Fig. 1:**
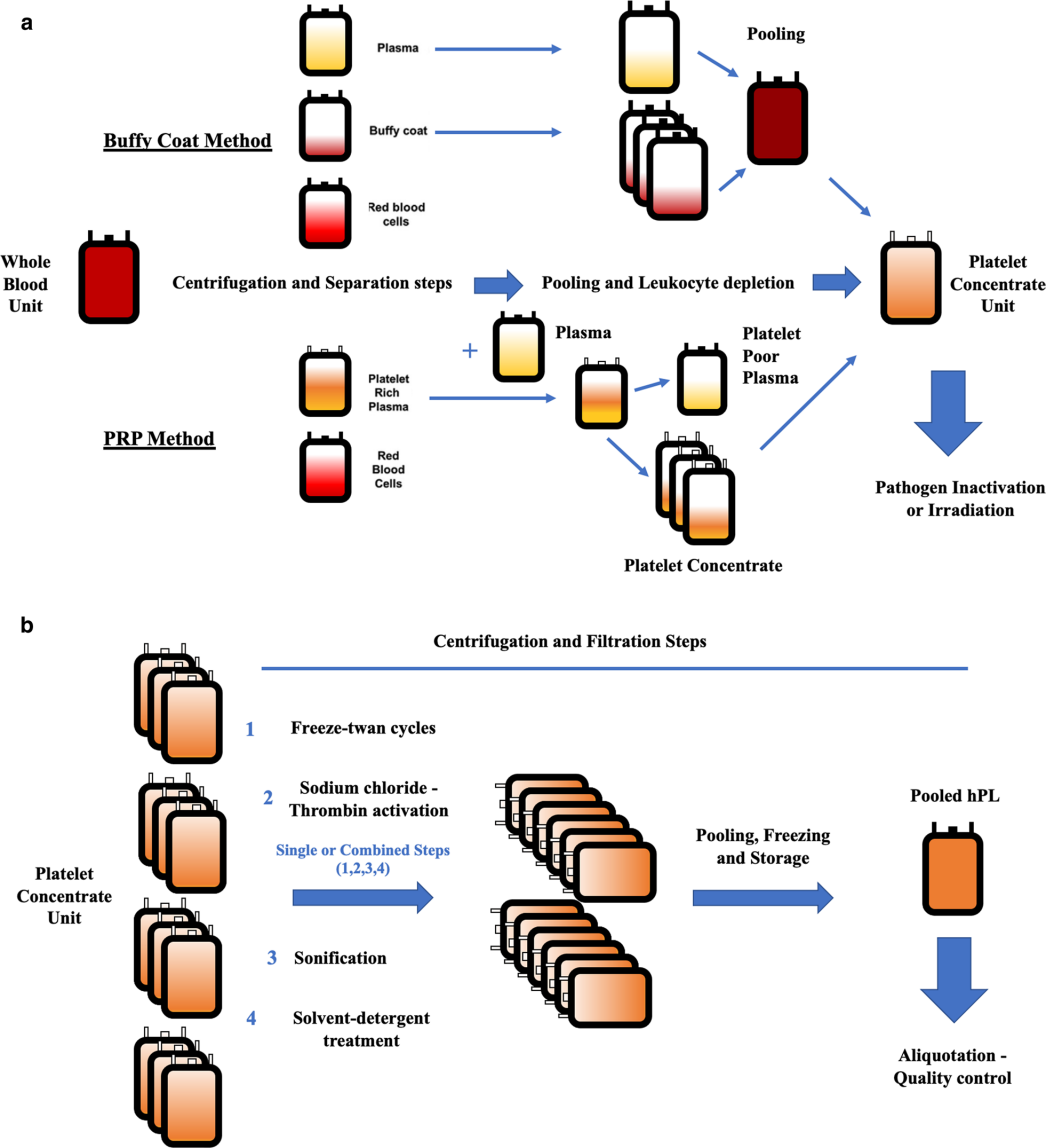
**A**, **B** Platelet concentrate units (PC) are prepared from anticoagulated whole blood mainly with buffy coat method or the PRP method. For the first method the whole blood unit is centrifuged and four buffy coat units plus one plasma unit (4 donors) are mixed together (final volume around 300 mL). Following a second centrifugation round the PC is leukocyte depleted and stored. For the PRP method, 4 or 5 PC are pooled to obtain a therapeutic unit of approximately 200 mL. Leukocyte depletion and storage are the following steps. **A** Apheresis platelet machines can extract directly PC from donors (less frequent method used—not shown in **A**)

Effectively, despite the higher production costs than FBS, hPL, being a human-derived xenofree media supplement, is more frequently replacing FBS in in vitro cell culture, aiming for clinical application, abolishing the immunological and infectious concerns around FBS translationability. HPL showed properties in supporting and even enhancing MSC proliferation, in retaining stem cell phenotype and multilineage differentiation capacity [5].

Despite the growing use of hPL in vitro cell culture and the earnest interest for translational purposes, the implementation of hPL collides with the lack of adequate knowledge of its biosafety risks, composition (e.g. growth factors content) and its variability related to the multiple manufacturing steps [4].

Recent research is investigating the impact of different manufacturing methods of the lysate (Fig. 1B) on MSC expansion, gene expression and differentiation potential when hPL is applied for in vitro cell expansion [4].

In spite the potential greater safety of hPL when compared to FBS, pooling multiple donor samples (from 2 up to 52 donations with a final platelet concentration between 1 × 10^6^/mL and 15 × 10^9^/mL, according to different local legislation), to minimize donor diversities (less batch-to-batch heterogenicity against FBS) and achieve the required lot size to satisfy the high demands, could increase the risk of virus contamination. To minimize pathogen transmission, extended testing procedures on platelets concentrates, together with pathogen inactivation methods are essential requirements, which still need to be fully regulated. Finally, complete traceability of any health and quality issues of the batches will further reduce the transmission of diseases between donors and recipients and allow to follow up the efficacy.

In conclusion, the definition of univocal guidelines of hPL production and application in cell therapy are needed from both US Food and Drug Administration (FDA) and European Medicine Agency (EMA). Quality assessment of hPL should be guaranteed by stringent compliancy of national and international GMP regulations, specifically for cell therapy and cell-based pharmaceuticals.

Considering potential new frontiers in tissue engineering/cell therapy fields, the choice of a promising cell culture serum additive such as hPL deserves an open discussion focusing on the manufacturing standardization methods and biological safety before an extensive clinical translation.

To induce the rupture and release of the growth factors (GFs) contained in the platelets, PC undergoes to four different procedures: repeated freeze/thaw cycles (1), direct platelet activation with the addition of chloride calcium (CaCl_2_) or thrombin (2), sonification (3) and solvent/detergent (S/D) treatment (4). (1) One to 5 freeze/thaw cycles (frozen from − 30 °C to − 80 °C, thawed at 37 °C) is the most frequent and cost-effective lysis procedure. (2) CaCl_2_ induces thrombin activation, fibrin formation and platelet degranulation. Eventually, human or recombinant thrombin can be added with similar effect. (3) Rapid and economic method which can be combined with one or more freeze/thaw cycles: sonification for 30 min at 20 kHz represents the most common protocol applied to stimulate degranulation. (4) This method achieves with the same step the GFs release and the lipid-enveloped virus inactivation. Lastly, the lysates are pooled and stored (Fig. 1B).

## Data Availability

Not applicable.
